# Radiofrequency Ablation for Chronic Pain: Mechanistic Insights and Emerging Innovations

**DOI:** 10.7759/cureus.99056

**Published:** 2025-12-12

**Authors:** Courage O Idahor, Sarah Mokobia, Ndidiamaka Ogbonna, Gloria E Eguahon, Oyidia Edema, Chinonye Opene, Osamagbe Osaghae, Ekene Chinedu, Nosa J Oronsaye, Olamide Ogunfuwa, Olaide B Sulaiman, Jideofor C Okoye

**Affiliations:** 1 Emergency Medicine, Nottingham University Hospital NHS Trust, Nottingham, GBR; 2 Emergency Medicine, James Paget University Hospitals NHS Foundation Trust, Gorleston-on-Sea, GBR; 3 Family Medicine, Edo State University Iyamho, Iyamho, NGA; 4 Family Medicine, University of Benin, Benin, NGA; 5 Psychiatry, University of South Wales, Cardiff, GBR; 6 Family Medicine, All Saints University School of Medicine, Roseau, DMA; 7 Internal Medicine, University of Benin, Benin, NGA; 8 General Medicine, NHS Lanarkshire, Lanarkshire, GBR; 9 Internal Medicine, Wexham Park Hospital, London, GBR; 10 Emergency Medicine, Sandwell and West Birmingham NHS Foundation Trust, Birmingham, GBR; 11 Accident and Emergency, Mid Cheshire Hospitals NHS Foundation Trust, Crewe, GBR; 12 Medical Education, Mersey and West Lancashire Teaching Hospital NHS Trust, Lancashire, GBR

**Keywords:** ablative therapy, chronic pain management, interventional pain medicine, interventional pain therapy, pain modulation, pulsed radiofrequency nerve ablation, radiofrequency ablation (rfa)

## Abstract

Chronic pain remains a significant global health burden, with far-reaching consequences for individual well-being, societal productivity, and healthcare resources. Despite advances in pharmacological and interventional strategies, many patients experience inadequate relief or intolerable side effects, underscoring the need for alternative approaches. Radiofrequency ablation (RFA) has emerged as a minimally invasive, targeted therapy for a wide range of chronic pain syndromes, including spine-related disorders, peripheral neuropathic pain, complex regional pain syndrome (CRPS), and refractory cancer pain. This narrative review explores the historical evolution of RFA, tracing its origins from early neurosurgical experiments to its current place in contemporary pain medicine. Mechanistic insights into RFA highlight its ability to disrupt nociceptive pathways at both cellular and molecular levels, with biophysical principles guiding lesion formation and efficacy. The review examines conventional, pulsed, cooled, and bipolar RFA techniques, comparing their effectiveness and indications across pain syndromes. Recent technological innovations, such as advanced image guidance, neuro-navigation, robotic assistance, and regenerative hybrid strategies, are discussed as transformative trends poised to enhance the precision and outcomes of RFA. The safety profile, including immediate and delayed complications, is critically appraised alongside established preventive strategies and management protocols. Evidence from clinical trials, meta-analyses, and professional guidelines is synthesized to inform best practice, while also addressing ethical, legal, and accessibility considerations in diverse patient populations. Collectively, this review underscores the evolving role of RFA in chronic pain management, highlights persistent challenges, and calls for ongoing research to optimize patient selection, procedural safety, and equitable access worldwide.

## Introduction and background

Chronic pain is a pervasive global health challenge affecting millions of individuals worldwide [1]. Pain is generally considered chronic when it persists on most days, or every day, for at least three months [2,3]. Beyond discomfort, the severity of chronic pain can significantly impair physical function, diminish quality of life, and contribute to psychological distress [2,3]. Previous studies have estimated the global prevalence and incidence of chronic pain at approximately 20% and 10% of adults, respectively [1,4]. This means that about one in five people, or around 1.5 billion individuals, suffer from chronic pain, with an additional one in ten new cases diagnosed annually [5]. However, this burden is not evenly distributed [5-7]. The elderly and those living in low- and middle-income countries comprise a significant proportion of chronic pain sufferers [6,7]. The higher occurrence in these populations has been linked to the increased prevalence of chronic diseases, including cancer and HIV/AIDS [6-8].

In addition to its substantial toll on individual well-being, chronic pain imposes a considerable economic burden on healthcare systems. This economic impact is due both to direct medical costs and indirect losses from decreased productivity, absenteeism, and long-term disability [9]. Multiple studies have quantified the economic costs of chronic pain and pain-associated conditions. In the United States, people experiencing chronic pain incurred an extra $8068 per year in medical expenses and $2923 in lost productivity per individual compared with those without chronic pain. Overall, the total economic impact of chronic pain in 2021 was estimated at $722.8 billion, comprising $530.6 billion in healthcare costs and $192.2 billion in reduced work productivity [10]. Similarly, a Norwegian study estimated that chronic pain imposed an economic burden equivalent to 4% of the country's Gross Domestic Product (GDP) [11]. Among adolescents in the United States, Groenewald et al. found that chronic pain resulted in mean and median costs of $11787 and $6770 per adolescent, respectively, totaling about $19.5 billion annually for this population [12].

Despite the existence of several management modalities, chronic pain remains a significant clinical challenge. This challenge is multifactorial, resulting from the complexity and heterogeneity of pain syndromes, variable patient responses, and the biopsychosocial nature of pain [13]. Pharmacological treatments such as opioids and non-opioid analgesics, including nonsteroidal anti-inflammatory drugs (NSAIDs), anticonvulsants, and antidepressants, have long served as the cornerstone of chronic pain management [13-15]. Due to the risks of complications such as opioid use disorder and overdose, the 2022 Centers for Disease Control Practice Guideline for Prescribing Opioids for Pain recommends non-opioids as the first-line treatment for chronic pain. When opioids are considered, immediate-release formulations and the lowest effective dosage are strongly advocated [15].

Beyond pharmacological therapies, several interventional strategies such as nerve blocks, epidural steroid injections, and implantable devices have been developed as alternatives for chronic pain management [2,15]. However, these conventional therapies also have their shortcomings [2,16]. Adverse drug effects can significantly limit the use of pharmacological agents, sometimes making them contraindicated in certain populations [16]. NSAIDs, for example, are commonly associated with gastrointestinal problems such as dyspepsia, stomach aches, and diarrhea, and have also been linked to adverse cardiovascular, hepatic, and hematological effects [17,18]. The development of tolerance, dependence, and misuse of opioids further complicates their use for chronic pain [15]. Similarly, procedural and post-procedural complications are significant limitations of conventional non-pharmacological interventions [19].

In response to these limitations, there has been growing interest in minimally invasive targeted therapies capable of modulating pain pathways with greater precision and fewer systemic side effects. Among these, radiofrequency ablation (RFA) has emerged as a promising interventional modality. RFA involves the application of high-frequency alternating current through a needle electrode placed close to the target nerve, generating localized heat that disrupts pain signal transmission [20]. This technique has been effectively used to treat various chronic pain conditions, including facial pain, headaches, post-mastectomy pain, major joint pain, and musculoskeletal pain [21]. The primary advantage of RFA over pharmacological and conventional interventional therapies is its ability to offer longer-lasting pain relief with minimal invasiveness and a relatively low complication rate [22].

Since its inception in the early 1900s, RFA technologies have evolved from conventional thermal RFA to include pulsed RFA, cooled RFA, and water-cooled RFA, each with distinct mechanisms and clinical indications [23]. These innovations aim to enhance safety and reduce collateral tissue damage associated with RFA for chronic pain management [22,23]. However, despite the growing adoption of RFA in pain management, several questions remain, including those concerning its long-term efficacy, ideal patient selection criteria, and comparative effectiveness relative to other interventional and pharmacological therapies.

Given this evolving landscape, there is a need for a comprehensive review that synthesizes current knowledge on RFA for chronic pain management, highlights recent mechanistic discoveries, and examines technological innovations that may shape its future applications. This review provides an overview of the epidemiology and burden of chronic pain, critically evaluates the limitations of existing pharmacological and interventional treatment options, and summarizes the origin, timeline, and principles of RFA technologies. Furthermore, it explores the current evidence for the use of RFA in clinical trials and real-world practice, while identifying key challenges, controversies, and knowledge gaps that influence the use of RFA in chronic pain management.

## Review

Historical evolution of radiofrequency ablation in pain management

The concept of using heat to destroy tissues dates back centuries; however, the specific technique of RFA only emerged in the mid-20^th^ century [20]. Initially developed within the field of neurosurgery, Martin Kirschner, a German surgeon, is credited as the first to experiment with this technology for the treatment of trigeminal neuralgia (TN) in 1931 [22,24]. By applying RFA to the Gasserian ganglion, Kirschner demonstrated that continuous radiofrequency could create a focal thermal lesion along a neural pathway, effectively interrupting pain transmission and blocking nociception. Although this was a groundbreaking discovery, commercialisation of the technique did not occur until about thirty years later, when Aronow and Cosman developed the first commercially available radiofrequency systems [24].

By the 1970s, RFA had begun to attract broader interest in the management of chronic pain, particularly for spinal conditions [25]. One of the earliest standardized applications of this technology involved targeting the medial branches of the dorsal rami in the facet joints of the spine, an approach aimed at treating facet joint pain, a common source of chronic back pain [25]. Over time, with continued technological advancements, RFA has become increasingly integrated into interventional pain medicine [20,22].

The evolution of RFA from a basic lesioning technique to a sophisticated and widely used intervention for pain management has been driven by substantial technological progress. Early RFA systems were rudimentary, typically consisting of a monopolar electrode connected to a radiofrequency generator delivering high-frequency current. These devices relied heavily on manual temperature monitoring with minimal feedback control, making it difficult to create precise lesions. Furthermore, early systems could not differentiate between tissue types, increasing the risk of unintended nerve damage and limiting the effectiveness of pain relief. As clinical experience with RFA expanded, so did the understanding of lesion dynamics and tissue thermodynamics, fueling the push for more advanced RFA systems [20,22,23].

A key innovation in RFA technology was the introduction of temperature-controlled ablation and impedance-monitored generators. These systems enabled real-time monitoring and regulation of tissue temperature at the electrode tip, reducing the risk of tissue overheating, nerve charring, or collateral tissue damage [20,26]. Another major advancement was the development of cooled RFA, which represented a paradigm shift in lesion creation. In this technique, internally circulated water cools the electrode tip during energy delivery, preventing excessive heat accumulation at the tissue-electrode interface. This process maintains lower impedance and allows for the creation of larger, more uniform lesions [20].

Among the most novel innovations is pulsed radiofrequency (PRF), which provides a less destructive alternative to conventional RFA. Unlike continuous energy delivery that heats tissues to create lesions, PRF emits high-voltage bursts of energy interspersed with silent phases, keeping tissue temperatures below 42°C. This prevents structural nerve damage while still modulating pain pathways. Due to its high precision, PRF has been particularly useful in treating neuropathic pain conditions and in ablating nerves situated near sensitive structures, such as the dorsal root ganglion or trigeminal nerve [20].

Beyond these core advancements, other emerging RFA technologies continue to improve efficacy, safety, precision, and target selectivity in chronic pain management [20]. Progress has also been made in the design of probes and cannulas. Modern electrodes now come in a variety of shapes and sizes, from curved and steerable tips to multi-tined or cluster probes, enhancing the precision of targeted lesion placement and improving procedural manoeuvrability [23]. Additionally, the integration of imaging guidance has greatly improved procedural accuracy, with modalities such as fluoroscopy, ultrasound, and CT-based navigation systems now commonly used during RFA procedures [27,28].

Since its first use in 1931, RFA has achieved significant milestones. Before the 1960s, the technology was mainly limited to the treatment of TN. However, beginning in the 1960s, physicians began using RFA for spinal conditions, targeting sympathetic chains and peripheral nerves. In 1975, Dr. C. Norman Shealy published one of the earliest clinical protocols for facet joint denervation using medial branch RFA. The ongoing evolution of RFA technology led to the commercial availability of generators with improved temperature regulation in 1981. By the late 1980s, protocols for lumbar facet neurotomy had become standardized, and further technological developments continued. In 1997, Sluijter and Cosman introduced PRF, opening new avenues for neuromodulation in neuropathic pain management [29].

The 2000s marked further expansion in the indications for RFA. In 2004, the technology gained approval for treating sacroiliac joint (SIJ) pain, discogenic pain, and peripheral joint osteoarthritis. Between 2006 and 2010, clinical trials increasingly supported the use of RFA for chronic knee pain [29,30]. Research has even shown RFA to be superior to steroid injections for certain spine-related pain conditions, while its effectiveness in non-spine-related conditions has also been demonstrated [29,30]. These include knee osteoarthritis via genicular nerve ablation, migraines via sphenopalatine ganglion ablation, and lower back pain via cluneal nerve ablation [29]. Current studies are exploring innovative approaches, including artificial intelligence-enhanced targeting and biomarker-driven patient selection, further expanding the possibilities for RFA in pain management [31].

Mechanistic basis of radiofrequency ablation

Peripheral and central neurons involved in the pain pathway possess a sophisticated capacity to detect and respond to a variety of extracellular stimuli, such as pro-inflammatory mediators and neurotransmitters. This is made possible through the expression of cell surface receptors that rapidly initiate intracellular signaling cascades. Following injury or infection, activation of cell surface G protein-coupled receptors (GPCRs) triggers complex signaling processes. These processes generate action potentials in neurons and stimulate inflammatory responses, including cytokine secretion by immune cells [32]. However, it is now recognized that events occurring at the cell surface alone may not fully account for the signaling repertoire of these receptors. After an initial wave of cell surface signaling, activated GPCRs can interact with endocytic proteins like the adaptor protein β-arrestin (βArr), facilitating clathrin-mediated internalization. Traditionally, βArr-mediated internalization was thought to terminate GPCR signaling. Yet, for several GPCRs implicated in pain, research has demonstrated that endocytosis can promote a distinct "second wave" of intracellular signaling, emanating from endosomes and Golgi membranes, which is both spatially and temporally unique compared to initial surface events [32].

Central sensitization is a key mechanism contributing to chronic pain states. It refers to the process by which the central nervous system develops a heightened state of excitability, amplifying the processing of nociceptive (pain) signals. Numerous mechanisms underlie central sensitization, including alterations in glutamatergic neurotransmission, NMDA receptor-mediated hypersensitivity, loss of inhibitory controls (disinhibition), and complex glial-neuronal interactions [33]. In glutamate/NMDA receptor-mediated sensitization, persistent injury or intense stimulation leads C and Aδ nociceptors to release neurotransmitters such as glutamate, substance P, calcitonin gene-related peptide (CGRP), and ATP onto neurons in lamina I of the superficial dorsal horn. Under these conditions, previously silent NMDA glutamate receptors on postsynaptic neurons become activated, resulting in increased intracellular calcium and subsequent activation of a range of calcium-dependent signaling pathways and second messengers, including mitogen-activated protein kinase (MAPK), protein kinase C (PKC), protein kinase A (PKA), phosphatidylinositol 3-kinase (PI3K), and Src kinase. This cascade increases the excitability of output neurons and facilitates the enhanced transmission of pain messages to the brain [33].

PRF interventions operate through cellular and molecular mechanisms that can be broadly categorized into effects on nociceptive signalling, immune activity, and synaptic function. PRF modulates various biological pathways involved in chronic neuropathic pain (neuralgia) [34]. In terms of nociceptive signaling, PRF influences ion channels (such as Na/K ATPase, HCN, and P2X3), neuropeptides like CGRP, neurotransmitters (aspartate, citrulline, M-ENK, and glutamate), postsynaptic receptors (AMPA-R and GABA-B), and synaptic proteins (KCC2). PRF also exerts important effects on immune activity, altering microglial markers (CD3, CD56, and Iba1), inflammatory cytokines (IL-6, IL-17, IRF8, IFN-γ, and TNFα), and several intracellular proteins implicated in immune-mediated neuropathic pain, including BDNF, β-catenin, JNK, p38, and ERK1/2 [34]. Through these mechanisms, PRF can modulate both neuronal and immune components of pain processing, contributing to its efficacy in the management of chronic pain syndromes.

RFA itself is a well-established technique for inducing thermally mediated coagulation necrosis in targeted tissues, either via percutaneous approaches using image guidance or direct surgical placement of thin electrodes [35,36]. The extent of coagulation necrosis generated by conventional monopolar radiofrequency electrodes depends on several factors, including the total energy delivered, duration of radiofrequency application, and the length and gauge of the electrode tip [37]. These parameters are carefully optimized to maximize lesion effectiveness while minimizing collateral tissue damage.

RFA is now increasingly utilized in the management of chronic pain conditions. The standard RFA technique involves applying an alternating current at the catheter tip, which produces localized heating and leads to ablation of the targeted tissue substrate [38]. While high temperatures at the catheter tip (>70°C) and within the tissue are effective for ablation, they also increase the risk of complications such as coagulum formation and steam pops, which can limit safe power application. The advent of irrigated RFA catheters has addressed some of these limitations, allowing greater energy transfer to tissue and enabling the creation of larger, more controlled lesions. Alternatively, cryoablation, which involves cooling tissues to -80°C, produces dense, circumscribed scars through the formation of intra- and extracellular ice crystals, and the cryomapping process enhances procedural safety [38].

Conventional RF ablation delivers high temperatures, up to 80°C, via a probe that emits a strong alternating electrical field for 60 to 90 seconds [39]. The resulting thermal degradation denatures proteins in the targeted nerve tissue, effectively interrupting pain signal transmission. Cooled RFA, a refinement of the technique, has also shown clinical effectiveness by disrupting deep sensory nerves and thereby interfering with pain pathways [40]. Through these diverse and increasingly sophisticated mechanisms, RFA offers a powerful approach for modulating pain at both peripheral and central levels, underpinned by advances in our understanding of neural, synaptic, and immune contributions to chronic pain syndromes.

Clinical applications across chronic pain syndromes

The SIJ is a critical axial joint connecting the sacrum to the pelvis. Inflammation around this joint, resulting from factors such as trauma, pregnancy, or prior lumbar fusion, can lead to significant pain and disability [41]. Sacroiliac (SI) joint dysfunction is widely recognised as a common source of low back pain, with prevalence estimates suggesting that up to 25% of low back pain cases may be attributed to SIJ pain [42-44]. While conservative management remains the initial approach, many patients experience persistent symptoms that require interventional options such as cooled RFA [42]. Recent global evidence indicates that cooled RFA targeting the sacral nerves supplying the SI joints provides superior and more sustained pain relief compared to traditional treatment modalities for SI joint dysfunction [41].

The management of SIJ dysfunction using RFA involves the application of radio waves to generate an electric current that heats and ablates the nerve fibers, ultimately reducing pain sensation. The current standard approach utilizes unipolar radiofrequency lesioning of the dorsal rami lateral branch nerves that innervate the SI joint [45]. This method has demonstrated promising outcomes, with approximately 60% of patients achieving sustained pain relief at six months post-procedure. Among the variations of radiofrequency interventions, PRF differs from conventional RFA by delivering high-voltage bursts in short intervals, allowing heat dissipation during "silent" phases and preventing the formation of thermal lesions. Consequently, PRF does not destroy nerve tissue, offering a less destructive alternative [45]. Various mechanisms have been proposed to explain the analgesic effects of PRF, including modulation of the c-Fos pathway by alternating electric fields and alteration of the transcription factor ATF3, which affects cellular stress in C and Aδ pain fibers. Although the precise mechanisms are still under investigation, the short-term pain relief achieved with PRF is notable [45,46].

Facet syndrome is another important contributor to chronic back pain, defined as pain originating from any structure of the intervertebral joint, including the fibrous capsule, synovial membrane, hyaline cartilage, or bone. Its prevalence varies between 5% and 15% of patients with low back pain, and some estimates suggest it could be as high as 30% in certain populations [47]. Osteoarthritis and degenerative changes are the most common underlying causes, particularly in older adults, while trauma and repetitive stress also play significant roles in the development of facet syndrome [47].

Minimally invasive surgical techniques for facet joint denervation have become increasingly important in the management of this condition [47]. Three main protocols are employed in radiofrequency facet denervation: conventional RFA, pulsed RFA, and RFA with cooling. Each technique has distinct technical features and clinical indications, allowing tailored approaches based on patient characteristics and the specific pain syndrome [47].

Neuropathic pain, which arises from primary damage or dysfunction of the nervous system, is another challenging pain syndrome often addressed by RFA. The pathogenesis of neuropathic pain is complex, involving changes in ion channel function, abnormal action potential generation and propagation, and both central and peripheral sensitization [48]. To date, RFA of peripheral nerves remains one of the simplest and most effective interventional approaches for neuropathic pain syndromes [48]. TN, for instance, is a severe orofacial pain condition that can be profoundly disabling [49]. While first-line therapy typically involves anticonvulsant medications, some patients require surgical intervention due to medication intolerance or contraindications to general anesthesia. In these cases, percutaneous techniques, including glycerol injections and RFA, offer effective alternatives. Numerous studies have demonstrated the efficacy of RFA in achieving significant pain relief for TN [49].

Occipital neuralgia is another example of neuropathic pain, characterized by persistent pain affecting the posterior scalp. It is often difficult to distinguish from other types of headaches [50]. For patients who are refractory to conservative management, RFA or occipital nerve stimulation has shown promise in providing durable relief [50].

Complex regional pain syndrome (CRPS) is a multifaceted disorder marked by sensory, motor, vasomotor, sudomotor, and trophic changes [51]. CRPS often develops as a complication following surgery or trauma, though spontaneous cases have been reported [51]. The International Association for the Study of Pain defines CRPS as a group of painful conditions that typically develop distally after trauma and are characterized by a greater intensity and duration of pain than would be expected from the original injury, frequently resulting in significant motor impairment [51]. While no universally accepted treatment protocol exists, RFA of the stellate ganglion has shown promise for long-term sympathetic suppression and pain relief in CRPS patients [51].

Refractory cancer pain remains a particularly challenging and distressing symptom for many patients [52]. RFA has emerged as an effective option for managing intractable pain caused by osseous metastases, especially in cases where pharmacological therapies have failed [52]. In recent years, minimally invasive therapies such as RFA have become an integral part of palliative care, offering effective pain control with a favorable safety profile [53]. Experimental and clinical studies have demonstrated that RFA, especially when used in combination with other treatments, can act synergistically to provide enhanced pain relief for cancer-related pain. With advances in technology, improved patient selection, and expanded clinical indications, RFA is now being considered as both a stand-alone and complementary therapy for primary and secondary malignancies, including those in high-risk populations [53]. Percutaneous RFA is increasingly used for cancer pain management, particularly in patients with bone metastases, and palliative debulking of tumor masses with RFA has been associated with substantial pain relief [53].

Variants of radiofrequency ablation techniques

The development and application of RFA in chronic pain management have evolved significantly, resulting in multiple technical variants designed to address the complexities and heterogeneity of pain syndromes. A comprehensive understanding of the main types of RFA techniques, their underlying principles, comparative clinical effectiveness, and specific indications is essential for optimizing patient outcomes in contemporary interventional pain medicine. Conventional RFA remains the foundational technique in interventional pain procedures. First developed in the mid-20th century, conventional RFA uses a high-frequency alternating current (typically 300-500 kHz) delivered through a monopolar electrode placed adjacent to the target neural structure [54]. The current generates localized tissue heating at the electrode tip, resulting in temperatures between 60°C and 90°C for a set duration, commonly 60 to 120 seconds [55]. This thermal lesion induces irreversible coagulative necrosis of neural tissue, effectively disrupting pain signal transmission. Conventional RFA is characterized by its focal lesion geometry, with lesion size and shape influenced by factors such as electrode gauge, exposed tip length, tissue impedance, and application time [56]. One of the primary advantages of conventional RFA is its established safety and efficacy in the treatment of chronic facet-mediated spinal pain, SIJ dysfunction, and certain peripheral neuralgias [57]. Multiple randomized controlled trials and systematic reviews have confirmed that conventional RFA provides significant and sustained pain relief for well-selected patients, often lasting six to twelve months or longer, with relatively low complication rates [58,59]. However, the technique requires precise electrode placement and is susceptible to limitations such as small lesion size, potential for missed target nerves due to anatomical variability, and the risk of thermal injury to adjacent non-target tissues [60].

In contrast to the continuous high-temperature lesioning of conventional RFA, PRF is designed to deliver short bursts of high-voltage energy interspersed with silent periods, maintaining tissue temperatures below the neurodestructive threshold, typically less than 42°C [61]. PRF was introduced in the late 1990s as a less destructive alternative, intended to modulate pain signaling pathways without causing overt nerve damage. The underlying mechanisms of PRF are complex and not fully elucidated, but emerging evidence suggests that PRF induces a series of cellular and molecular effects, including changes in gene expression, neurotransmitter release, and neuroinflammatory mediators, as well as alterations in synaptic transmission [62]. The clinical rationale for PRF is particularly compelling in neuropathic pain syndromes, where preservation of neural integrity is desirable, or in anatomically sensitive regions where destructive ablation could pose unacceptable risk [63]. Clinical studies have shown that PRF can provide significant pain relief in conditions such as TN, occipital neuralgia, dorsal root ganglion-mediated pain, and peripheral nerve entrapments, with fewer adverse events related to numbness or deafferentation [64,65]. Despite these advantages, the duration of analgesia with PRF may be shorter than with conventional RFA, and its efficacy in non-neuropathic pain syndromes is less well established [66].

Cooled RFA represents a significant innovation, designed to address some of the limitations associated with small or imprecise lesion formation in conventional RFA. In cooled RFA, a specialized electrode is internally perfused with circulating fluid (typically normal saline), which actively cools the electrode tip during energy delivery [67]. This process enables higher energy deposition into surrounding tissue while preventing excessive charring or desiccation at the probe-tissue interface. The result is a larger, more spherical lesion that extends further from the electrode, thereby improving the likelihood of encompassing anatomically variable neural branches [68]. Cooled RFA has been most extensively studied in the management of chronic knee osteoarthritis, SIJ pain, and lumbar facetogenic pain, with evidence demonstrating improved procedural success rates and longer-lasting pain relief compared to conventional RFA in some populations [69,70]. Additionally, the controlled thermal profile of cooled RFA reduces the risk of tissue overheating, charring, or inadvertent collateral damage to adjacent structures. Some studies have also reported favorable outcomes with cooled RFA for peripheral nerve and discogenic pain, although high-quality comparative data remain limited [71].

Bipolar and water-cooled radiofrequency techniques represent further refinements intended to optimize lesion geometry, efficiency, and safety. In bipolar RFA, two active electrodes are positioned in proximity, allowing current to pass directly between them rather than between a single electrode and a distant ground pad. This configuration generates a more predictable, elongated lesion that can be particularly advantageous in ablating long or diffuse nerve segments, such as in certain peripheral neuralgias or discogenic pain [72]. Water-cooled bipolar systems combine both the cooling and dual-electrode features to achieve extensive and homogeneous lesions while maintaining safe thermal limits [73]. These approaches may reduce the number of probe re-positionings required, potentially shortening procedural time and increasing the chance of complete denervation in complex anatomical landscapes.

A robust body of literature has investigated the comparative effectiveness and clinical indications for each of these RFA variants. Multiple randomized controlled trials and meta-analyses have established that conventional RFA is effective in reducing pain and improving function in lumbar facet syndrome, with effect sizes generally superior to placebo or sham procedures and often comparable to surgical options for selected patients [58,74]. Cooled RFA has demonstrated superior or at least non-inferior results to conventional RFA in the treatment of knee osteoarthritis, SIJ dysfunction, and chronic back pain, especially in populations with challenging or variable nerve anatomy [69,75]. A systematic review by Davis et al. concluded that cooled RFA provided higher rates of pain relief and longer duration of benefit in knee osteoarthritis compared to standard RFA, with a similar safety profile [76]. In the context of discogenic pain, bipolar and cooled RFA approaches have shown promise, with preliminary data suggesting better outcomes than conventional monopolar techniques, though further studies are warranted [77,78].

PRF, while initially viewed with scepticism due to its non-destructive nature, has gained increasing acceptance as a valuable modality in neuropathic and radicular pain. PRF appears to offer a favorable risk-benefit profile in cases where destructive ablation is contraindicated or undesirable, including trigeminal and occipital neuralgia, postsurgical neuropathies, and pediatric pain syndromes [63,65,79]. Nonetheless, systematic reviews highlight that PRF may have a more modest effect size and shorter duration of analgesia compared to conventional RFA for purely nociceptive pain conditions [66,80].

Selection of the appropriate RFA technique requires careful consideration of pain aetiology, anatomical characteristics, patient comorbidities, and prior response to interventions. For example, conventional RFA remains the gold standard for lumbar facet-mediated pain, while cooled RFA may be preferred in large joint or anatomically variable targets such as the SIJ and knee [69,74]. Bipolar and water-cooled systems are increasingly considered for discogenic or diffuse peripheral neuralgias, whereas PRF is favored in settings where neural preservation is critical or when treating complex neuropathic conditions [65,72].

Importantly, safety profiles differ among RFA variants. Conventional and cooled RFA techniques may be associated with higher rates of sensory loss, numbness, or post-ablation neuritis, though these are usually transient and rarely result in long-term morbidity [60,68]. PRF is generally well tolerated, with a lower incidence of adverse effects but also less consistent long-term relief in some patient groups [61,80]. Advanced techniques such as water-cooled and bipolar RFA are associated with a low incidence of serious complications when performed with proper imaging guidance and adherence to safety protocols [73,78]. The choice of RFA variant should be individualized, drawing upon evidence-based guidelines, anatomical understanding, and clinical judgment to maximize efficacy and minimize risk. As technology advances and further high-quality comparative studies emerge, the indications for each technique will likely continue to expand, ultimately improving patient outcomes and quality of life in chronic pain populations.

Innovations and future directions

The field of RFA for chronic pain has undergone a remarkable technological transformation over the past two decades. This evolution is now shaping a new era of precision pain medicine in which the boundaries between traditional ablation, neuromodulation, imaging, and even regenerative therapies are increasingly blurred. The relentless pursuit of improved safety, accuracy, and long-term efficacy has driven a wave of innovations that are changing the way clinicians select patients, target nerves, guide procedures, and conceptualize chronic pain management. As RFA shifts from a purely ablative approach to a component of integrated, multimodal strategies, its future appears tightly linked to ongoing advances in imaging, neuro-navigation, robotics, biomarker science, and regenerative medicine.

Recent developments in image guidance have been at the heart of improvements in the precision and safety of RFA procedures. Traditionally, fluoroscopy has served as the gold standard for needle placement in spinal and peripheral nerve RFA due to its real-time visualization of bony landmarks and rapid workflow. However, fluoroscopy alone has limitations, particularly in soft tissue contrast and in the context of aberrant anatomy or altered post-surgical landscapes. The adoption of high-resolution ultrasound has introduced significant benefits, especially for peripheral nerve procedures and in pediatric or thin patients, by enabling direct visualization of nerves, blood vessels, and surrounding soft tissues. Ultrasound guidance not only reduces radiation exposure but also allows dynamic assessment of needle-nerve relationships and can be invaluable in complex anatomies or previously operated regions [81,82]. In certain scenarios, particularly deep or small targets, computed tomography (CT) guidance can offer unparalleled spatial resolution, facilitating precise lesioning in anatomically challenging cases such as SIJ pain or tumors involving the vertebral body [83,84]. The advent of hybrid imaging systems that combine real-time ultrasound with fluoroscopic overlays is enabling clinicians to harness the advantages of both modalities, further refining procedural accuracy and minimizing complications [85,86].

The integration of neuro-navigation and robotic-assisted technologies into interventional pain medicine represents another frontier in RFA's evolution. Computer-assisted navigation systems, initially developed for neurosurgery and orthopedic oncology, are now being adapted for spinal and peripheral nerve RFA, leveraging preoperative imaging and real-time tracking to guide the operator with sub-millimeter accuracy [87,88]. These systems not only facilitate reproducible and efficient probe placement but can also enhance safety by mapping critical structures and optimizing trajectories, thereby reducing the risk of nerve or vascular injury. Robotic-assisted RFA, although still in early clinical phases, has shown promise in both experimental and early clinical studies. Robotic arms, under the control of the interventionalist, are capable of executing complex trajectories and maintaining steady positioning that surpasses human dexterity, particularly during prolonged or multi-lesion ablations [89]. These innovations are especially relevant in deep-seated or anatomically variable targets, and they have the potential to reduce operator fatigue, procedural time, and variability in clinical outcomes [90,91].

The intersection of RFA with neuromodulation and pharmacogenomics is also opening new avenues for chronic pain management. Traditional RFA induces neurodestruction and interrupts pain transmission, but an increasing body of research is exploring the modulation of pain pathways through sub-ablative, pulsed, or targeted approaches. The combination of RFA with spinal cord stimulation or peripheral nerve stimulation is being investigated as a strategy to provide both immediate and long-lasting pain relief, potentially synergizing the benefits of ablation and neuromodulation [92,93]. In parallel, pharmacogenomic profiling is emerging as a tool to personalize pain management by predicting individual responses to analgesic medications and interventions. Studies have begun to identify genetic polymorphisms associated with opioid metabolism, inflammatory pathways, and even pain perception, laying the groundwork for biomarker-driven selection of candidates most likely to benefit from RFA or its combination with other therapies [94,95]. While these approaches are still in the translational stage, their future integration into clinical practice could optimize treatment efficacy, reduce adverse events, and foster more individualized patient care.

Biomarker-guided patient selection is poised to be a transformative force in refining indications for RFA. Historically, the selection of candidates for RFA has been based largely on clinical evaluation, imaging findings, and diagnostic nerve blocks. However, the heterogeneity of chronic pain syndromes and inter-individual differences in response have highlighted the need for more objective and predictive markers. Recent advances in molecular pain biology have identified a range of candidate biomarkers, including neuropeptides (such as substance P and CGRP), cytokine profiles, and neuroimaging-derived markers, that may correlate with pain phenotypes and predict treatment outcomes [96,97]. For example, functional MRI and positron emission tomography (PET) imaging can assess changes in regional brain activation patterns before and after RFA, offering a window into central pain processing and potentially enabling the stratification of responders and non-responders [98,99]. In the future, integration of molecular, genetic, and imaging biomarkers into routine practice could help tailor RFA approaches to the individual patient's pain biology, thereby maximizing efficacy and minimizing unnecessary interventions [100].

A particularly exciting innovation in the future of RFA for chronic pain is the emergence of regenerative and hybrid approaches. Traditionally, RFA has been viewed as a destructive technique, but recent studies suggest that it may also have modulatory and even regenerative effects on neural and perineural tissues, especially when used in combination with biologics. One area of growing interest is the combination of RFA with platelet-rich plasma (PRP) or stem cell therapies, which aim to promote tissue repair and modulate inflammatory responses following ablation [101,102]. Animal studies and early human trials have shown that the application of PRP or mesenchymal stem cells after RFA can enhance nerve regeneration, reduce perineural fibrosis, and improve functional outcomes in chronic pain conditions such as osteoarthritis and neuropathic pain [103,104]. These hybrid procedures are believed to harness the anti-inflammatory and trophic properties of biologics while benefiting from the immediate pain-relieving effects of ablation. Additionally, advances in biomaterials and drug-delivery systems are being explored to provide local, sustained release of analgesic or regenerative agents directly at the ablation site, potentially extending the duration of pain relief and reducing systemic side effects [105,106].

Artificial intelligence (AI) and machine learning are emerging as important enablers of innovation in interventional pain procedures. These technologies are being developed to automate image segmentation, optimize probe placement, predict lesion size, and even guide real-time adjustments during the RFA procedure, thereby reducing operator dependence and inter-observer variability [107]. The use of AI-driven decision support systems can also assist clinicians in patient selection, risk stratification, and outcomes prediction, further personalizing chronic pain management [108,109]. As these technologies become more robust and accessible, their integration with imaging, robotics, and electronic health records is likely to redefine the workflow and scope of RFA in clinical practice.

Despite these promising innovations, several challenges remain. The translation of advanced imaging, navigation, biomarker, and regenerative strategies from research to routine practice will require rigorous validation through multicenter clinical trials, standardisation of protocols, and attention to cost-effectiveness. The future of RFA for chronic pain management is likely to be characterised by increasing precision, integration of multimodal approaches, and an emphasis on patient-specific solutions. As the field continues to evolve, ongoing collaboration between clinicians, engineers, and basic scientists will be essential to realize the full potential of these innovations and ensure their safe, effective, and equitable application.

Safety profile and complications

RFA has become a cornerstone in the interventional management of chronic pain syndromes, appreciated for its ability to provide targeted and durable relief when conservative therapies fail. However, as with any invasive procedure, RFA carries the potential for immediate and delayed complications. Understanding these risks, the underlying anatomical and procedural contributors, and the protocols for prevention and management is crucial to optimizing patient outcomes and maintaining safety in clinical practice.

Complications following RFA can be broadly classified into immediate and delayed events, each with its own clinical implications and management strategies. Immediate complications typically occur intraoperatively or within hours of the procedure. These include bleeding at the puncture site, hematoma formation, vascular injury, inadvertent thermal damage to non-target tissues, transient neuritis, and allergic reactions to anesthetics or contrast agents [110,111]. Among the most frequently reported acute adverse events is pain exacerbation at the treatment site, sometimes termed "post-ablation flare." This transient increase in pain is generally attributed to local inflammation or nerve irritation and often resolves with conservative management [112]. Another recognised risk is infection, which can range from superficial cellulitis at the entry site to deeper soft tissue or even epidural abscess, though the overall incidence remains low in settings that adhere to sterile technique [113,114].

Thermal injury is an inherent risk due to the mechanism of RFA, which relies on the generation of heat to induce coagulative necrosis in neural tissue. If the ablation probe is positioned too close to adjacent structures such as the skin, muscle, bowel, or large vessels, unintentional collateral damage can occur. Such injuries may manifest as cutaneous burns, muscle necrosis, bowel perforation, or even vascular compromise [115,116]. The likelihood of these complications is influenced by procedural factors, including probe type, lesion size, power settings, duration of ablation, and real-time monitoring accuracy. Meticulous technique and appropriate imaging guidance can significantly mitigate these risks [117,118].

Delayed complications can present days to weeks after the procedure and may include neuritis, deafferentation pain, sensory or motor deficits, and, in rare cases, neuroma formation. Neuritis is characterised by burning, shooting, or dysesthetic pain in the distribution of the ablated nerve, sometimes with paresthesia or numbness. While typically self-limiting, neuritis can persist for several weeks and occasionally requires pharmacologic intervention with neuropathic pain agents or corticosteroids [119,120]. Deafferentation pain, a phenomenon where disruption of sensory nerves results in paradoxical pain, is infrequently encountered but can be especially challenging to treat [121]. Permanent sensory or motor deficits are exceedingly rare, particularly when established protocols for patient selection, probe placement, and stimulation testing are followed [122]. In the context of spinal procedures, there is a remote but important risk of epidural or subarachnoid haemorrhage, especially in patients with coagulopathy or on anticoagulation therapy [123].

The anatomical landscape in which RFA is performed introduces unique risk factors. Certain nerve targets, such as those near the dorsal root ganglion, sympathetic chain, or close to major vascular structures, require heightened caution. The proximity of the ablation field to neural elements responsible for motor function increases the risk of inadvertent motor impairment, making precise electrode positioning and motor testing imperative [124]. Additionally, the anatomical variability of nerve pathways, especially in the cervical and thoracic spine, can complicate safe access and increase the likelihood of non-target injury [125,126]. Previous surgeries, radiation therapy, or congenital anomalies may further distort landmarks and elevate procedural complexity [127].

Procedural risk factors encompass both technical and patient-related variables. High-energy settings or prolonged ablation times may increase the radius of thermal spread and heighten the chance of collateral damage [128]. Lack of adequate imaging guidance, such as performing RFA without real-time fluoroscopy, ultrasound, or CT, is associated with greater complication rates, particularly in anatomically challenging regions [129,130]. Patient comorbidities, including diabetes mellitus, immunosuppression, and poor skin integrity, can predispose to wound complications, delayed healing, and infection [131]. Use of anticoagulant or antiplatelet medications is a well-known risk factor for post-procedural bleeding and mandates careful periprocedural planning [132].

To minimise the risk of complications, several preventive strategies are recommended as best practice. A comprehensive pre-procedural evaluation is fundamental and should include assessment of anatomical landmarks, vascular structures, prior surgical alterations, and patient comorbidities [133]. Laboratory investigations, such as coagulation studies, may be warranted in high-risk individuals. Adherence to strict aseptic technique, including skin preparation and sterile draping, remains the cornerstone of infection prevention [134]. Real-time imaging guidance using fluoroscopy, ultrasound, or CT is strongly advocated to ensure precise needle placement and to monitor proximity to critical structures [135]. Pre-ablation sensory and motor stimulation testing can help confirm accurate targeting and avoid injury to motor fibres, particularly in procedures involving the spine or major plexuses [136].

Technical refinements have also contributed to improved safety profiles. The use of cooled RFA probes can produce larger but more controlled lesions with lower peak temperatures at the probe tip, thereby reducing the risk of charring, tissue desiccation, and non-target thermal injury [137]. PRF offers another safety advantage by delivering sub-ablative bursts of energy, minimizing neural destruction and reducing the incidence of persistent neuritis or deafferentation pain [138]. Robotic and neuro-navigational assistance, although still in early stages for routine pain procedures, promise to further enhance accuracy, reduce operator fatigue, and decrease complication rates in complex cases [113,139].

Management protocols for RFA complications are tailored to the type and severity of the event. For mild post-ablation flare or neuritis, conservative measures such as NSAIDs, local ice, and rest are usually sufficient. More severe or persistent neuropathic symptoms may benefit from adjunctive therapies, including gabapentinoids, tricyclic antidepressants, or short courses of corticosteroids [120,140]. Infections require prompt evaluation and initiation of antibiotics, and abscess formation may necessitate drainage [114]. Hematomas that are small and stable may be managed expectantly, but expanding or compressive hematomas, especially in the vicinity of neural structures, warrant urgent intervention [123]. For cases involving thermal injury to adjacent organs or tissues, multidisciplinary consultation with surgical or wound care specialists may be necessary. Motor deficits or suspected nerve injury should prompt immediate imaging and neurologic assessment to guide further management [141].

The long-term safety of RFA is supported by numerous studies demonstrating low rates of major complications when procedures are performed by experienced practitioners using standardized protocols [142,143]. The recurrence of pain, often due to nerve regeneration or disease progression, is a recognised limitation rather than a direct complication, though repeat procedures are generally well tolerated with similar safety profiles [144]. Patient education and informed consent, addressing the spectrum of potential risks and realistic expectations for outcome, are essential components of safe practice.

Evidence-based clinical guidelines

Studies have consistently demonstrated the effectiveness of RFA across multiple medical specialties, with ongoing research expanding its applications and refining procedural techniques. The evidence base has grown substantially in the past decade, focusing increasingly on long-term outcomes and comparative effectiveness studies. Meta-analyses have shown that RFA achieves 89% complete ablation rates for breast cancer and a 96% good-to-excellent cosmetic result [145]. However, in hepatocellular carcinoma, surgical resection offers higher chances of long-term survival, though RFA is associated with lower complication rates [145]. Long-term cohort studies, including over 10-year follow-up data for thyroid microcarcinoma, conclude that RFA is both effective and safe in this setting [146]. Major randomized trials have compared different RFA techniques for atrial fibrillation, with no significant difference observed between the methods studied regarding overall safety [147]. Recent meta-analyses also confirm the effectiveness of RFA for uterine fibroids and pancreatic neuroendocrine tumors [148]. Globally, there has been a steady increase in the recognition and adoption of RFA in the treatment of cardiac arrhythmias, with its minimally invasive approach and rapid technological advancements enhancing both safety and efficacy [149].

The past decade has also seen significant progress in evidence-based guidelines for RFA procedures, with numerous professional societies contributing detailed recommendations for clinical practice. Notably, the American Society of Pain and Neuroscience (ASPN) published the "Latest Evidence-Based Application for Radiofrequency Neurotomy (LEARN): Best Practice Guidelines" in September 2021 [150]. These guidelines represented a multidisciplinary effort to examine evidence-based medicine across various anatomical applications of radiofrequency neurotomy, including cervical, thoracic, and lumbar spine procedures, as well as peripheral joint interventions. In 2023, ASPN published specialized guidelines for radiofrequency ablative procedures in patients with implanted devices in response to the growing complexity of pain management in this population [151]. These guidelines addressed critical safety considerations for patients with cardiac implantable electronic devices, spinal cord stimulators, and other implanted technologies, emphasizing electromagnetic interference mitigation and procedural modifications to enhance safety during radiofrequency procedures. The American Society of Regional Anaesthesia and Pain Medicine has also played an active role in advancing evidence-based practice through ongoing literature reviews and clinical guidance publications. A comprehensive 2024 ASRA review examined the evidence for diagnostic blocks before RFA of lumbar facet joint innervation, highlighting ongoing debates regarding diagnostic block requirements [152]. The International Pain and Spine Intervention Society and many healthcare insurers in the United States continue to recommend two sets of diagnostic medial branch blocks as the most reliable method for identifying facet joint pathology before proceeding with RFA [152]. Furthermore, the International Spine Intervention Society has continually served as a foundational resource through its practice guidelines for spinal diagnostic and treatment procedures, which provide detailed technical specifications for percutaneous radiofrequency procedures [153]. These guidelines have been widely referenced and adopted across professional societies, establishing standardized approaches to spinal radiofrequency interventions.

Key themes have emerged across society's guidelines. There is ongoing discussion regarding optimal diagnostic block protocols, with evidence supporting both single and dual diagnostic block paradigms depending on clinical circumstances and insurance requirements [152]. The importance of contrast injection during diagnostic blocks is emphasized to ensure proper needle placement and avoid intravascular injection [154]. The emergence of cooled radiofrequency technology has also been incorporated into recent guidelines, recognizing its ability to create larger thermal lesions compared to traditional neurotomy, which is particularly beneficial for peripheral joint applications [150]. Safety protocols have evolved significantly, with current guidelines emphasizing comprehensive pre-procedural assessment, motor and sensory testing before ablation, maintaining minimal sedation to ensure patient communication, and specific risk mitigation strategies for patients with implanted medical devices [151]. These developments reflect the impressive growth of RFA from an emerging technique to an established interventional pain management modality with robust evidence-based standards.

The most significant controversy in RFA concerns diagnostic block protocols before treatment. Multiple unresolved issues persist regarding what constitutes optimal patient selection, including differing opinions on what defines a positive diagnostic block, the number of blocks required, the level of pain relief considered significant, the injectate used, and the timing for evaluating outcomes of both diagnostic blocks and RFA [152]. The International Pain and Spine Intervention Society and most US healthcare insurers advocate for dual diagnostic medial branch blocks as the most reliable method for identifying facet joint pathology before RFA [152]. However, this approach presents a cost-effectiveness paradox, as some studies suggest that proceeding directly to radiofrequency denervation without diagnostic blocks is the most cost-effective paradigm [152]. This discrepancy between clinical guidelines and economic analyses remains unresolved. Another striking paradox is seen in lumbar RFA practice patterns. Utilization continues to rise despite controversial efficacy data. While some studies demonstrate significant, durable improvements in pain and functional limitation, others show limited benefit, highlighting a disconnect between evidence and practice patterns [155]. Notably, lumbar RFA utilization increased by approximately 131% between 2007 and 2016 [155].

Technical and procedural controversies persist regarding optimal RFA parameters and techniques. In the treatment of TN, research indicates that different temperatures in radiofrequency therapy may lead to different outcomes, while the efficacy of PRF for pain relief in this setting remains debated [156]. Higher temperatures are associated with complications such as facial numbness and other sensory deficits [156]. There is also ongoing mechanistic uncertainty. Currently, no clear evidence demonstrates pain pathway disruption in response to high-frequency electrical current alone. It is thought that both electrical and thermal effects contribute to clinical benefits, but this remains to be fully elucidated [110]. This uncertainty complicates efforts to optimize treatment parameters and predict outcomes. Hardware compatibility is another area of debate. The safety and efficacy of RFA in patients with implanted medical devices have historically been questioned due to theoretical risks of hardware heating [157]. Recent research has challenged these concerns, showing equivalent outcomes in patients with and without hardware, but definitive guidelines are still lacking [157]. Questions regarding long-term efficacy and durability remain. Systematic reviews indicate persistent uncertainty about RFA's long-term effectiveness across pain conditions. While studies on lumbar facet and SIJs generally show significant short-term pain reduction, the evidence for discogenic low back pain is mixed, and researchers call for future studies to examine long-term clinical significance [158]. International research also reveals significant outcome variability driven by patient population differences, procedural techniques, and healthcare infrastructures [149]. Despite widespread adoption, RFA continues to face challenges, including complications, patient selection, and long-term efficacy [149]. Finally, there is a lack of procedural standardization. Surveys reveal considerable variation in clinical practice, with no standardized protocol for treating knee pain via genicular nerve block and ablation, emphasizing the need for future research to establish best practices [159]. These controversies highlight the need for high-quality, standardized research to establish evidence-based protocols for RFA across all anatomical applications, with priority given to consensus diagnostic criteria, procedural techniques, long-term outcome measures, and guidelines for special populations such as those with implanted devices.

Ethical, legal, and accessibility considerations

The application of RFA in the management of chronic pain is rapidly expanding, but its clinical integration brings forth significant ethical, legal, and accessibility challenges. Addressing these issues is critical to ensuring that RFA is implemented in a manner that is both effective and just, while protecting patient rights, managing societal resources, and promoting health equity. The ethical obligations of clinicians, regulatory frameworks, and healthcare system capacities all intersect as RFA becomes more prominent in pain medicine.

A fundamental ethical and legal requirement in the use of RFA is obtaining robust informed consent and ensuring that patient expectations are managed realistically. Informed consent is more than a signed document; it represents a process of transparent dialogue between patient and provider that clarifies the risks, benefits, alternatives, and potential outcomes of the procedure [160]. Given the evolving landscape of RFA, clinicians must articulate not only the intended benefits but also the uncertainties regarding long-term efficacy, the possibility of recurrence of pain, and the risk of adverse events such as nerve injury, infection, or transient neuritis [110]. Patient-centered communication is particularly important in chronic pain syndromes, where psychological, social, and functional aspects often complicate patient understanding and expectations [161]. Failure to communicate the limits of RFA or to recognize patient vulnerabilities may undermine trust and could be construed as a breach of duty, potentially exposing practitioners to legal liability [122]. Shared decision-making models have thus been advocated, empowering patients to take an active role in their care, weigh procedural risks, and better align treatment with their values and goals [162,163].

Another dimension of ethical practice relates to the equity of access to RFA in underserved regions and populations. Despite its minimally invasive nature and demonstrated clinical benefits, RFA remains inaccessible for many individuals, particularly those living in low- and middle-income countries, rural areas, and marginalized communities [135,164]. Disparities in access arise from limited numbers of trained interventional pain specialists, insufficient imaging or operating room infrastructure, and financial constraints, including lack of insurance coverage or prohibitive out-of-pocket costs [165,166]. In addition, patients in these settings may experience delays in diagnosis, limited health literacy, or cultural barriers to the acceptance of interventional pain procedures [167]. This inequity is concerning, especially given the disproportionate burden of chronic pain among disadvantaged groups, and risks reinforcing existing health disparities [112]. Global health organizations and pain societies have highlighted the need to prioritize training, infrastructure investment, and advocacy for broader coverage of pain interventions, including RFA, to ensure that innovation does not further entrench inequalities [168,169].

Cost-effectiveness and healthcare policy implications are increasingly important considerations as RFAs' use expands. High-quality evidence supports the efficacy of RFA for conditions such as lumbar facet joint pain, SIJ dysfunction, and knee osteoarthritis, yet its adoption at scale must be balanced against the direct and indirect costs to healthcare systems [158]. Economic evaluations suggest that while the upfront cost of RFA is greater than that of conservative management or corticosteroid injections, its durability and potential to reduce reliance on opioids, avoid surgery, and decrease disability may result in net cost savings over time [69,70]. A systematic review found that RFA for lumbar facet joint pain was cost-effective in terms of quality-adjusted life years gained, especially when compared to repeated conservative treatments [170]. However, cost-effectiveness varies by indication, healthcare setting, and patient population, and may be undermined by high rates of recurrence or suboptimal patient selection [122].

Healthcare policy frameworks shape the accessibility and utilization of RFA. In some high-income countries, RFA is reimbursed by public and private insurers for select indications, while in others, restrictive coverage criteria or a lack of explicit guidelines pose barriers to patient access [171,172]. Policymakers must weigh the evidence for RFA's clinical benefit, patient preference, and system sustainability when designing coverage policies and clinical pathways [133]. Emerging health technology assessment processes increasingly incorporate not only clinical and economic evidence but also ethical and equity considerations in decision-making [173]. There is a growing consensus that broadening access to RFA and other advanced pain interventions should be linked to robust patient registries, post-market surveillance, and periodic reappraisal of coverage decisions as new evidence accumulates [129].

Beyond system-level policies, there are legal considerations around practitioner competence, procedural safety, and liability. Regulatory bodies require that clinicians performing RFA possess adequate training, maintain certification, and adhere to evidence-based protocols [57]. Failure to follow standard-of-care practices may result in professional sanctions or litigation, particularly if complications are deemed preventable or if informed consent was inadequate [122,174]. Clear documentation of procedural planning, patient counselling, and follow-up care is essential both for ethical practice and legal protection.

The expansion of RFA also prompts ongoing debate about resource allocation and the potential for technology-driven disparities in pain management. There is an ethical imperative to ensure that advances in pain care are not limited to privileged populations but are leveraged to reduce suffering broadly and equitably [168,175]. Efforts to develop lower-cost technologies, expand provider training, and implement telemedicine-supported pain interventions may help to bridge access gaps, especially in remote or resource-limited settings [176,177].

## Conclusions

RFA stands at the forefront of interventional strategies for chronic pain, offering effective and lasting relief for patients refractory to conventional treatments. Its mechanistic basis, diverse technique variants, and broad clinical applicability make it an invaluable tool in pain medicine. Advances in imaging, robotics, and biomarker science are driving the field toward even greater precision and individualized care, while regenerative and hybrid approaches hint at a future of more holistic pain modulation. However, challenges remain regarding procedural safety, long-term outcomes, and equitable access, particularly in underserved regions. Rigorous research, ongoing technological refinement, and inclusive health policy are essential to realizing RFA's full potential. Ultimately, the integration of mechanistic insights, innovative technology, and patient-centered practice will shape the next chapter of RFA in chronic pain management, ensuring its benefits reach those most in need.
